# Pain score, desire for pain treatment and effect on pain satisfaction in the emergency department: a prospective, observational study

**DOI:** 10.1186/s12873-018-0189-y

**Published:** 2018-11-08

**Authors:** Judith E. van Zanden, Susanne Wagenaar, Jozine M. ter Maaten, Jan C. ter Maaten, Jack J. M. Ligtenberg

**Affiliations:** 1 Department of Emergency Medicine, University of Groningen, University Medical Center Groningen, Groningen, The Netherlands; 2 Department of Cardiology, University of Groningen, University Medical Center Groningen, Groningen, The Netherlands

**Keywords:** Pain management, Emergency department, Oligoanalgesia, Pain satisfaction, Numeric rating scale

## Abstract

**Background:**

Pain management in the Emergency Department has often been described as inadequate, despite proven benefits of pain treatment protocols. The aim of this study was to investigate the effectiveness of our current pain protocol on pain score and patient satisfaction whilst taking the patients’ wishes for analgesia into account.

**Methods:**

We conducted a 10-day prospective observational study in the Emergency Department. Demographics, pain characteristics, Numeric Rating Scale pain scores and the desire for analgesics were noted upon arrival at the Emergency Department. A second Numeric Rating Scale pain score and the level of patient satisfaction were noted 75–90 min after receiving analgesics. Student T-tests, Mann-Whitney U tests and Kruskall-Wallis tests were used to compare outcomes between patients desiring vs. not desiring analgesics or patients receiving vs. not receiving analgesics. Univariate and multivariate logistic regression models were used to investigate associations between potential predictors and outcomes.

**Results:**

In this study 334 patients in pain were enrolled, of which 43.7% desired analgesics. Initial pain score was the only significant predictive factor for desiring analgesia, and differed between patients desiring (7.01) and not desiring analgesics (5.14). Patients receiving analgesics (52.1%) had a greater decrease in pain score than patients who did not receive analgesics (2.41 vs. 0.94). Within the group that did not receive analgesics there was no difference in satisfaction score between patients desiring and not desiring analgesics (7.48 vs. 7.54). Patients receiving analgesics expressed a higher satisfaction score than patients not receiving analgesics (8.10 vs. 7.53).

**Conclusions:**

This study pointed out that more than half of the patients in pain entering the Emergency Department did not desire analgesics. In patients receiving analgesics, our pain protocol has shown to adequately treat pain, leading to a higher satisfaction for emergency health-care at discharge. This study emphasizes the importance of questioning pain score and desire for analgesics to prevent incorrect conclusions of inadequate pain management, as described in previous studies.

## Background

As pain is a common complaint of patients visiting the Emergency Department (ED) [1], its early recognition and appropriate treatment is important. Experience of pain is one of the criteria on which quality of care in the ED is reviewed and a key factor in preventing unintentional, avoidable harm.

Since Marks and Sachar published a landmark paper about undertreatment of pain in the ED [2], this became a frequently investigated subject. The benefits of pain treatment protocols have been proven [3], yet pain management in the ED has often been described as inadequate [1, 4, 5]. This raises the question whether undertreatment of pain is caused by inadequate adherence to pain protocols, or, that ‘oligoanalgesia’ – a term to describe the phenomenon of undertreatment of pain – is overstated, as Green postulated in the Annals of Emergency Medicine [6]. Another question is whether pain should always be treated, given the fact that not all patients in pain desire analgesics [7, 8]. To our knowledge, a prospective study on the effects of protocolized pain treatment on pain score and patient satisfaction, while taking desire for analgesics into account, has not been previously published.

The main goal of our study was to prospectively investigate the effectiveness of our current pain protocol on pain score and patient satisfaction in our ED; the desire for analgesia by the patient is hereby taken into account. ‘Effectiveness’ of pain treatment was defined as a decrease in pain score and increased level of satisfaction with care. A pain score reduction of > 2 Numeric Rating Scale (NRS) was considered clinically relevant [9, 10]. Secondary goals were to evaluate arguments for refusing analgesics and reasons for not receiving analgesics, despite patients’ expressed desire for pain relief. We hypothesized that patients receiving analgesics would have a stronger decrease in pain score and a higher score of patient satisfaction compared to patients who did not receive analgesics.

## Methods

**Fig. 1:**
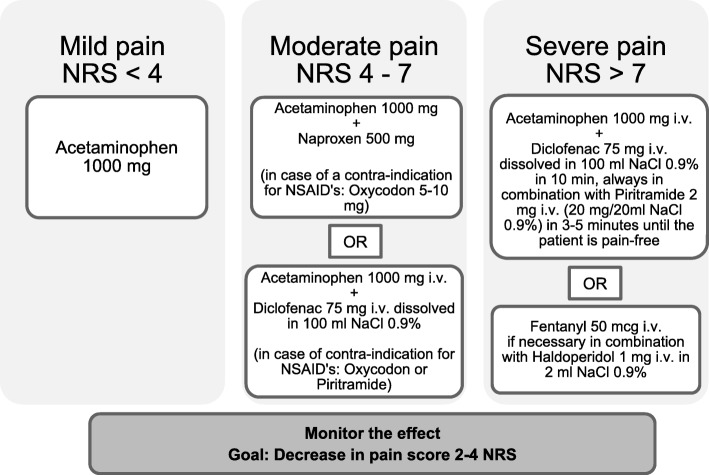
The University Medical Center Groningen pain protocol

This study had a prospective, observational design and was conducted at the ED of the University Medical Center Groningen (UMCG). The ED of this tertiary teaching hospital has an annual volume of approximately 32.000 patients, of which around 22.000 are estimated to be non-trauma patients. At the onset of the study, an analgesic protocol was present in the ED (Fig. 1). Data was obtained by study personnel filling out a comprehensive questionnaire for patients > 18 years old visiting the ED. This study was approved by the institutional review board of the UMCG. All patients signed the informed consent to participate in this study.

All patients with complaints of recent pain (< 3 months) were questioned. Exclusion criteria were patients < 18 years, no recent pain (< 3 months), patients in a life-threatening situation, patients with impaired mental status or neuropsychiatric diseases leading to inability to fill in the questionnaire (e.g. patients suffering from delirium, dementia or psychosis), a language barrier or absence of informed consent.

We used a manual questionnaire for a continuous period of 10 days, 24 h a day, to collect data for every patient visiting the ED.

During their stay, all patients were questioned twice. Immediately after triage of the patient, data was collected on demographics (age, sex), triage colour code (Table 1), allergy for analgesics, medical specialty of referral, pain characteristics (location, duration, type and character of pain) and pain score at that time, using the Numeric Rating Scale, ranging from 0 (no pain) to 10 (worst pain the patient can imagine). We checked whether the patient had already received analgesics before arriving at the hospital, e.g. self-medication or analgesics given by the general physician or ambulance staff. Furthermore, we asked whether the patient desired pain management. In case there was no desire for analgesics, the patient was asked about the reason. The second part of the questionnaire was filled in 75–90 min after taking the initial questionnaire. First, we checked if the patient had received analgesics in the ED and the type and dose of analgesics given. An analgesic was defined as any drug prescribed with the aim to reduce pain, such as acetaminophen, nonsteroidal anti-inflammatory drugs (NSAID) and opioids, as described in the analgesic ladder of the World Health Organization (WHO) [11]. Besides that, in accordance with previous articles published on this subject, drugs having a possible indirect effect on pain level were also included. Those were nitroglycerin for chest pain, proton pump inhibitors (PPI) for abdominal pain and benzodiazepines [7, 8]. If the patient did not receive any analgesics but had previously indicated a desire to receive analgesics, we asked the patient and the nurse why analgesics were not administered. The patient was asked again to rate pain severity on the Numeric Rating Scale. Lastly, the patient was asked to express their satisfaction on how their entire pain management was executed, rated on a Numeric Rating Scale, ranging from 0 (very dissatisfied) to 10 (very satisfied).

**Table 1:** Triage colour code, as used in the University Medical Center Groningen

Urgency	Name	Colour	Maximum waiting time
1	Immediately	Red	0 min
2	Highly urgent	Orange	10 min
3	Urgent	Yellow	60 min
4	Standard	Green	120 min
5	Not urgent	Blue	240 min

Primary outcomes were change in pain score and patient satisfaction score. Secondary outcomes were reasons to refuse analgesics and identifying why patients did not receive analgesics, despite desire for pain relief.

After completion of the study, all blinded questionnaires were manually entered in SPSS and checked by two investigators.

### Statistical analysis

Binominal and categorical data are presented as frequencies of occurrence and percentages. Continuous variables are presented as means and standard deviations (SD). The student T-test or Mann-Whitney U test was used to compare means between patients desiring vs. not desiring analgesics or patients receiving vs. not receiving analgesics. To compare between multiple groups, the Kruskall-Wallis test was used, followed by post hoc Mann-Whitney U tests. *P*-values were described to demonstrate differences between groups, with *p* < 0.05 considered significant.

To investigate associations between potential predictors and outcomes, logistic regressions were conducted. Factors that showed to be significant in univariate logistic regressions were included in a multivariate logistic regression model. Associations are presented by odds ratios (OR) and confidence intervals (CI). *P*-values of < 0.05 were again considered significant. All analyses were performed using IBM SPSS Statistics version 22.

## Results

**Fig. 2:**
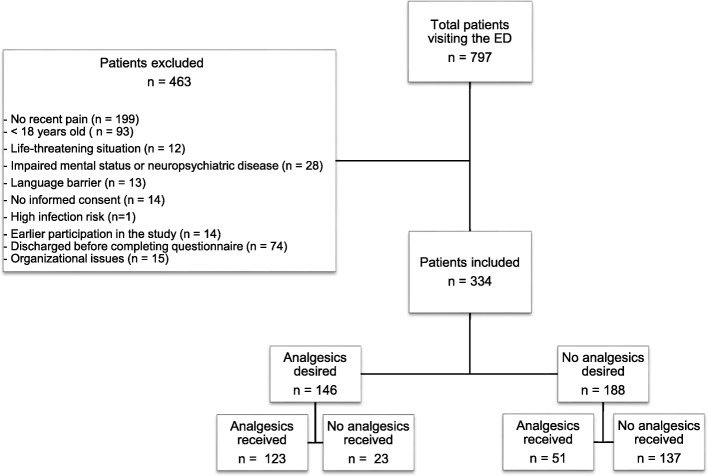
Consort diagram of patient disposition through the study

The total number of patients that visited the ED during the 10 days of inclusion was 797 (Fig. 2). Of those, 199 patients had no recent pain. Furthermore, 93 patients were excluded for being < 18 years old, 12 patients were excluded for being in a life-threatening situation, 28 patients had an altered mental status or neuropsychiatric disease, in 13 patients a language barrier was present, 14 patients did not give informed consent and 1 patient could not be questioned due to a too high infection risk. In addition, 14 patients were excluded because they participated earlier in the study, 74 patients were discharged before completing the questionnaire and 15 patients were not questioned because of organizational issues. In total, 463 patients were excluded from the study.

### Characteristics of study subjects

The total number of patients included during the 10-day study was 334. The mean age was 49 years and 53% were male. The overall mean initial pain score was 5.96 NRS (SD 2.28). Half of the patients had already taken analgesics before entering the ED (50.9%) and 5.1% of the patients indicated to have an allergy for an analgesic. Patient and pain characteristics are outlined in Table 2.

**Table 2:** Patient and pain characteristics with subdivision in desire for analgesics

Patient factors	Desire for analgesics	No desire for analgesics	p-value
	(*n* = 146)	(*n* = 188)	
Gender			
Male (*n* = 177)	72 (40.7%)	105 (59.3%)	0.235
Female (*n* = 157)	74 (47.1%)	83 (52.9%)	
Mean age (SD)	48.38 (18.44)	49.06 (18.39)	0.675
Triage colour code			
Blue (*n* = 7)	2 (28.6%)	5 (71.4%)	0.010
Green (*n* = 130)	45 (34.6%)	85 (65.4%)	
Yellow (*n* = 175)	84 (48%)	91 (52%)	
Orange (n = 18)	13 (72.2%)	5 (27.8%)	
Red (*n* = 4)	2 (50%)	2 (50%)	
Specialty of referral			
Traumatology (*n* = 78)	38 (48.7%)	40 (51.3%)	0.121
Internal medicine (*n* = 50)	22 (44%)	28 (56%)	
Emergency medicine (*n* = 26)	12 (46.2%)	14 (53.8%)	
Cardiology (*n* = 45)	12 (26.7%)	33 (73.3%)	
Abdominal surgery (*n* = 32)	17 (53.1%)	15 (46.9%)	
Orthopedics (*n* = 32)	9 (28.1%)	23 (71.9%)	
Lung diseases (*n* = 16)	7 (43.8%)	9 (56.3%)	
Neurology (n = 15)	9 (60%)	6 (40%)	
Other (*n* = 40)	20 (50%)	20 (50%)	
Pain location			
Upper extremities (*n* = 47)	18 (38.3%)	29 (61.7%)	0.033
Lower extremities (*n* = 54)	23 (42.6%)	31 (57.4%)	
Abdomen (*n* = 75)	42 (56%)	33 (44%)	
Head/face (*n* = 24)	13 (54.2%)	11 (45.8%)	
Back (*n* = 12)	7 (58.3%)	5 (41.7%)	
Chest (n = 54)	14 (25.9%)	40 (74.1%)	
Neck (n = 3)	1 (33.3%)	2 (66.7%)	
More than 1 location (n = 47)	23 (48.9%)	24 (51.1%)	
Other (n = 18)	5 (27.8%)	13 (72.2%)	
Pain duration			
< 2 h (*n* = 68)	32 (47.1%)	36 (52.9%)	0.047
2–6 h (*n* = 52)	27 (51.9%)	25 (48.1%)	
6–12 h (*n* = 31)	19 (61.3%)	12 (38.7%)	
12 h – 3 days (n = 78)	32 (41.0%)	46 (59.0%)	
> 3 days (*n* = 105)	36 (34.3%)	69 (65.7%)	
Pain pattern			
Constant (*n* = 259)	122 (47.1%)	137 (52.9%)	0.020
Intermittent (n = 75)	24 (32%)	51 (68%)	
Pain character			
Sharp (*n* = 121)	58 (47.9%)	63 (52.1%)	0.425
Pressing (*n* = 66)	27 (40.9%)	39 (59.1%)	
Nagging (*n* = 62)	31 (50%)	31 (50%)	
Burning (n = 18)	5 (27.8%)	13 (72.2%)	
Cramping (n = 7)	3 (42.9%)	4 (57.1%)	
More than 1 answer (*n* = 48)	19 (39.6%)	29 (60.4%)	
Other (n = 12)	3 (25%)	9 (75%)	
Mean initial pain score (SD)	7.01 (1.94)	5.14 (2.19)	0.029
Pre-ED analgesics			
Yes (*n* = 170)	83 (48.8%)	87 (51.2%)	0.055
No (*n* = 164)	63 (38.4%)	101 (61.6%)	
Analgesia allergy			
Yes (n = 17)	8 (47.1%)	9 (52.9%)	0.775
No (*n* = 317)	138 (43.5%)	179 (56.5%)	

P-values show differences of the whole factor group in relation to desire vs. no desire for analgesia

### Effect of pain protocol on pain score

Of the 334 enrolled patients, 146 (43.7%) patients desired analgesics upon arrival at the ED. Initial pain scores were higher in patients with a desire for analgesics compared to patients who did not wish analgesics (NRS score 7.01 [95% CI 6.70–7.33] vs. NRS score 5.14 [95% CI 4.82–5.45], Table 2). Desire for analgesics did not depend on factors such as gender, age, specialty of referral, pain character, pre-ED analgesics or known analgesic allergy. The variables triage colour code, pain location, pain duration and pain pattern (constant or intermittent pain) showed significant differences between the two groups (Table 2). Patients assigned to a group with a more urgent colour code were more likely to desire analgesics and patients with chest pain were less likely to desire analgesics. The duration of pain showed a trend towards medium pain duration (6–12 h) and a desire for analgesics. Patients suffering from constant pain were more likely to desire analgesics compared to patients experiencing intermittent pain (OR 1.89 [95% CI 1.10–3.26]).

The factors triage colour code, pain location, pain pattern and mean initial pain score were univariately significantly associated with the desire for analgesics and were the eligible factors for the multivariate model. In multivariate analysis, only the initial pain score was a significant predictive factor for desiring analgesia (Table 3).

**Table 3:** Factors associated with a desire for analgesics (univariate and multivariate analysis)

	Univariate analysis		Multivariate analysis	
Variable	OR (95% CI)	p-value	OR (95% CI)	p-value
Gender		0.236		
Male	Reference			
Female	1.30 (0,84–2.01)			
Age	0.10 (0.99–1.01)	0.737		
Triage colour code		0.021		0.139
Blue	0.40 (0.03–5.15)		1.23 (0.07–0.76)	
Green	0.53 (0.72–3.89)		1.65 (0.19–4.61)	
Yellow	0.92 (0.13–6.70)		2.98 (0.34–5.83)	
Orange	2.60 (0.28–3.81)		6.05 (0.54–8.08)	
Red	Reference		Reference	
Specialty of referral		0.14		
Traumatology	Reference			
Internal medicine	0,83 (0.41–1.69)			
Emergency medicine	0.90 (0.37–2.20)			
Cardiology	0.38 (0.17–0.85)			
Abdominal surgery	1.19 (0.52–2.72)			
Orthopedics	0.41 (0.17–1.00)			
Lung diseases	0.82 (0.28–2.42)			
Neurology	1.58 (0.51–4.86)			
Other	1.05 (0.49–2.26)			
Pain location		0.040		0.078
Upper extremities	Reference		Reference	
Lower extremities	1.20 (0.54–2.66)		1.29 (0.53–3.09)	
Abdomen	2.05 (0.97–4.32)		1.68 (0.74–3.79)	
Head/face	1.90 (0.70–5.15)		2.94 (0.97–8.93)	
Back	2.26 (0.62–8.19)		1.99 (0.48–8.21)	
Chest	0.56 (0.24–1.31)		0.68 (0.27–1.70)	
Neck	0.81 (0.07–9.54)		0.45 (0.03–6.67)	
More than 1 location	1.54 (0.68–3.51)		2.29 (0.92–5.71)	
Other	0.62 (0.19–2.03)		0.62 (0.17–2.21)	
Pain duration		0.051		
< 2 h	1.70 (0.91–3.18)			
2–6 h	2.07 (1.05–4.07)			
6–12 h	3.04 (1.33–6.94)			
12 h – 3 days	1.33 (0.73–2.44)			
> 3 days	Reference			
Pain pattern		0.021		0.314
Constant	1.89 (1.10–3.26)		1.38 (0.74–2.58)	
Intermittent	Reference		Reference	
Pain character		0.447		
Sharp	Reference			
Pressing	0.75 (0.41–1.38)			
Nagging	1.09 (0.59–2.00)			
Burning	0.42 (0.14–1.24)			
Cramping	0.82 (0.18–3.80)			
More than 1 answer	0.71 (0.36–1.40)			
Other	0.36 (0.08–1.40)			
Mean initial pain score	1.54 (1.36–1.74)	0.000	1.56 (1.37–1.77)	0.000
Pre-ED analgesics		0.056		
No	Reference			
Yes	1.53 (0.99–2.36)			
Analgesia allergy		0.775		
No	Reference			
Yes	1.15 (0.43–3.07)			

Of all the patients in pain, 174 patients (52.1%) received analgesics. The most commonly prescribed analgesics were acetaminophen (50.2%), NSAID’s (13.9%) and opioids (21.2%) (Table 4).

**Table 4:** Overview of prescribed types of analgesics

Administered drug	No. of patients (*n* = 174)
Acetaminophen	64 (36.8%)
NSAID	4 (2.3%)
Opioid	19 (10.9%)
Acetaminophen + NSAID	23 (13.2%)
Acetaminophen + NSAID + opioid	4 (2.3%)
Acetaminophen + opioid	25 (14.4%)
NSAID + opoid	1 (0.6%)
Nitroglycerin	9 (5.1%)
Benzodiazepine	1 (0.6%)
Others	24 (13.8%)

The number of patients receiving analgesics was greater than the number of patients who initially desired analgesics (174 vs. 146 patients). Of the 188 patients that initially refused analgesics, 51 patients had required and received analgesics at the time of the second questionnaire. Of the patients initially desiring analgesics, 23 patients did not receive them (Fig. 2). In order to accurately present the results, those two groups were temporarily excluded. In total, 260 patients (123 patients that desired and received analgesics and 137 patients that refused and did not receive analgesics) were included in the following analyses. Patients who received analgesics during their stay in the ED had significantly higher initial pain scores than those who did not receive analgesics (NRS score 7.09 [95% CI 6.76–7.42] vs. NRS score 4.82 [95% CI 4.46–5.19]). Pain scores measured 75–90 min after arrival decreased significantly in patients who received analgesics, with a reduction of 2.41 NRS (95% CI 2.02–2.79). The pain score of patients who received analgesics decreased more than patients who did not receive analgesics; the group of patients not receiving analgesics showed a reduction in pain score of 0.94 NRS ([95% CI 0.62–1.25], Table 5).

**Table 5:** Reduction in pain score in patients receiving analgesics vs. patients not receiving analgesics

	Analgesics received (*n* = 123)	No analgesics received (*n* = 137)	*p*-value
Initial pain score (95% CI)	7.09 (6.76–7.42)	4.82 (4.46–5.19)	0.00
Pain score after 75–90 min (95% CI)	4.68 (4.23–5.14)	3.89 (3.46–4.32)	0.013
∆ Pain score (95% CI)	2.41 (2.02–2.79)	0.94 (0.62–1.25)	0.00

### Effect of the pain protocol on patient satisfaction

Overall, all patients included in this study expressed a relatively high satisfaction of 7.83 (95% CI 7.66–7.99) for pain management. The majority of patients (67.1%) was satisfied (patient satisfaction score between 6 and 8 NRS), and 25.7% of the patients were even highly satisfied (patient satisfaction score 9–10). Only 7.2% of all patients included were dissatisfied (patient satisfaction score < 6 NRS).

After only analyzing patients who received analgesics in compliance with their initial wish, results show that patients receiving analgesics were significantly more satisfied than patients who did not receive analgesics (satisfaction score 8.06 [95% CI 7.80–8.31 vs. 7.54 [95% CI 7.24–7.84]).

### Reasons for refusing analgesics

The most common reason for not wanting analgesics, even though in pain, was bearable pain (57.4%), wanting to know the diagnosis first (10%) and using analgesics before visiting the ED (8%).

### Reasons for not receiving analgesics and the effect on patient satisfaction levels

**Fig. 3:**
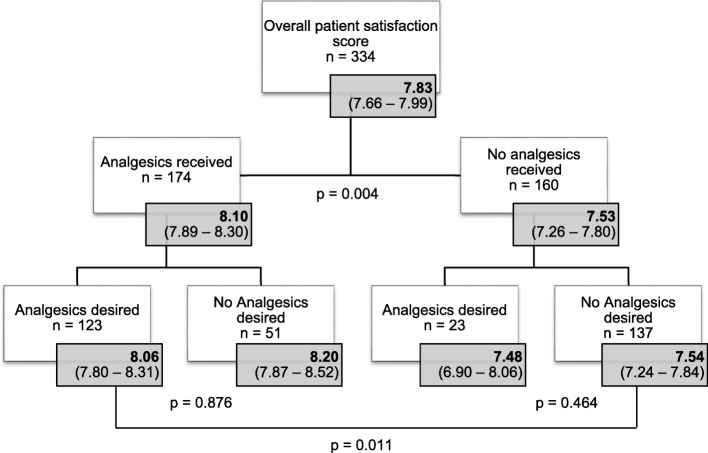
Patient satisfaction scores

As stated before, not all patients received analgesia according to their initial wish. Of the 146 patients desiring pain management, 23 patients did not receive analgesics. After performing subgroup analyses on patient satisfaction scores, results show that within the group that did not receive analgesics, patients who initially desired analgesics were not more dissatisfied than patients that did not desire analgesics (mean patient satisfaction score 7.48 [95% CI 6.90–8.06] vs. 7.54 [96% CI 7.24–7.84], Fig. 3). This result is important in investigating whether analgesics were incorrectly withheld from patients. In this cohort, the main reasons for not receiving analgesics included receiving an alternative non-pharmacological pain treatment such as splinting or applying ice or heat (31%), crowding in the ED (13%) and the clinical judgement of the nurse (13%). After correction for non-pharmacological pain treatment, 6.6% of the 334 patients in pain did not receive analgesics or a non-pharmacological pain treatment, despite their expressed desire.

## Discussion

Pain management is an important criterion of quality of care given in the ED and its adequacy has therefore been often discussed in literature.

According to our initial hypothesis, our study shows that the pain score in patients who received analgesics decreased more than in patients who did not receive analgesics. Patients who received analgesics were also significantly more satisfied than patients who did not receive analgesics. Those differences are in our study unlikely to be attributed to ‘undertreatment of pain’ in patients that did not receive analgesics. An important question in investigating ‘undertreatment of pain’ or ‘oligoanalgesia’ is whether all patients in pain desire analgesia. Previous research shows that nearly half of the patients in pain decline analgesia, which is in agreement with our results [7, 8]: of all the patients entering the ED in pain, less than half desired analgesics in our study. In those patients, ‘bearable pain’ was the most common reason for refusing analgesics. More urgent cases, medium pain duration of 6–12 h and constant pain were factors more associated with desiring analgesics, describing possible reasons why and when analgesics are desired.

Of patients that did not receive analgesics, the majority (86%) did not desire analgesics. Also, there was no difference in satisfaction between those patients and patients that did not receive analgesics despite their wish, suggesting that analgesics were not incorrectly withheld from patients. According to Allione et al., almost 10% of the patients do not receive analgesics despite their expressed desire [8], however, the reason for this was not described. Our study showed that receiving a non-pharmacological pain treatment was the most common reason for not receiving analgesics. After correcting for non-pharmacological pain treatment, only 6.6% of the patients did not receive analgesia or a non-pharmacological pain treatment despite their expressed desire for pain relief. Not correcting for the patients’ wish for analgesia might lead to low rates of analgesic administration and thus to incorrect conclusions with regard to inadequate pain management, reporting undertreatment percentages ranging from 20 to 50% as described before in literature [12].

Nevertheless, it has to be mentioned that despite the statistically significant differences, one might wonder if a difference of 0.52 in patient satisfaction score is clinically relevant. Previous studies have investigated the association between pain scores and patient satisfaction and stated that pain relief on itself is not the only factor affecting patient satisfaction [13, 14]. McNeill et al. showed a negative correlation between pain scores and patient satisfaction [15], while Svensson et al. showed a high patient satisfaction score despite high pain scores [16]. Phillips et al. concluded from their study that patient satisfaction should be addressed as an independent variable in assessing pain management [12].

Another important limitation of many earlier studies on ‘oligoanalgesia’ is their retrospective nature. Since pain perception is a subjective phenomenon, the presence or severity of pain should be determined by asking the patient at the moment of pain itself. Estimating the pain score, without asking the patient or asking the patient in retrospective, will not reliably reveal the real severity of pain [17]. Also, the number of patients receiving analgesics has more often been investigated, but rarely the actual effects of analgesics on lowering the pain score. A review of literature on oligoanalgesia stated that inconsistency and inadequacy of pain management is caused by multiple reasons including a lack of education in pain management, inadequate management programs to evaluate pain management, the clinicians’ attitude towards opioids and disbelief of pain reporting [18]. A generally accepted pain protocol is therefore important, providing guidelines aiming to prevent an inconsistency in pain treatment. Our study showed an overall pain reduction of 2.17 NRS in the group of patients treated with analgesia according to our pain protocol. Since a pain reduction of > 2 NRS is considered clinically relevant [9, 10], the current pain protocol can be considered adequate.

### Limitations of the study

Measuring severity of pain among ED patients can be challenging since pain perception is considered a subjective phenomenon. A retrospective way of investigating pain management will not provide a reliable picture [17]. However, a prospective way also has its limitations. The questionnaires were filled in by researchers questioning the patient. Some patients may have been inclined to provide responses based on an expectation of a correct response; thus skewing data. Secondly, the study was conducted under optimal circumstances of a working pain protocol. The presence of the researchers in the work field might have induced a ‘Hawthorne effect’, reminding nurses to pay more attention to pain management. Some patients expressed their desire for analgesics only to the researcher. In this case, the nurses were informed for ethical reasons. Further research will focus on the adherence to the pain management protocol under ordinary working circumstances and overcrowding.

An important challenge in investigating patient satisfaction on pain management was the subgroup of patients where no analgesics were given. For that reason, all patients were asked to rate the overall management routine regarding pain treatment in the Emergency Department, and not only the effect of the therapeutics itself. Nevertheless, it is possible that less attention was given to patients who initially did not desire analgesics, possibly causing the differences in satisfaction scores between the two groups. Patient satisfaction score was expressed as one overall grade in our questionnaire. Thus, specific reasons for expression of (dis)satisfaction regarding e.g. waiting time and communication, were not investigated.

This study is limited to a single, tertiary university hospital in Groningen, the north of the Netherlands. ED’s of all Dutch hospitals adhere to the same guidelines for pain management [19]. Therefore, pain protocols used in other Dutch hospitals can be considered as comparable. However, e.g. patient demographics can differ between ED’s. Our results should therefore not be generalized to other, dissimilar settings.

## Conclusions

In summary, our results show that less than half of the patients in pain entering the ED desire analgesics. Patients receiving analgesics were adequately treated according to the pain protocol, showing a clinically relevant reduction in pain score and higher satisfaction scores at discharge compared to patients who did not receive analgesics. Patients who did not receive analgesics despite their expressed wish were not incorrectly withheld from pain treatment, since satisfaction scores were comparable to patients who did not desire and did not receive analgesics. Additionally, most of the patients who did not receive pain medication despite desiring analgesics, received a non-pharmacological pain treatment. This study demonstrates that in this ED setting with a pain protocol in use, pain is adequately treated, and emphasizes the importance of taking the patients’ wish for analgesia into account when investigating ‘oligoanalgesia’ in the ED.

## Abbreviations

CI: Confidence Interval
ED: Emergency Department
NRS: Numeric Rating Scale
NSAID: Nonsteroidal Anti-Inflammatory Drugs
OR: Odds Ratio
PPI: Proton pump inhibitor
SD: Standard Deviation
UMCG: University Medical Center Groningen
WHO: World Health Organization
